# Self-Foldable Three-Dimensional Biointerfaces by Strain Engineering of Two-Dimensional Layered Materials on Polymers

**DOI:** 10.1021/acsami.4c17342

**Published:** 2025-01-29

**Authors:** Alonso Ingar Romero, Teodora Raicevic, George Al Boustani, Mrinalini Gupta, Ann-Caroline Heiler, Lukas Bichlmaier, Matteo Barbone, Markus Becherer, Daisuke Kiriya, Shigeyoshi Inoue, Joe Alexander, Kai Müller, Andreas R. Bausch, Bernhard Wolfrum, Tetsuhiko F. Teshima

**Affiliations:** 1 School of Computation, Information and Technology, Technical University of Munich, Garching 85748, Germany; 2 Medical & Health Informatics Laboratories, NTT Research Incorporated, Sunnyvale, California 94085, United States; 3 School of Natural Sciences, Technical University of Munich, Garching 85748, Germany; 4 Graduate School of Arts and Sciences, The University of Tokyo, Tokyo 153-8902, Japan; 5 Faculty of Science and Technology, Keio University, Yokohama, Kanagawa 223−8522, Japan

**Keywords:** self-assembly, strain engineering, lab-on-a-chip, two-dimensional layered materials, nanomembranes

## Abstract

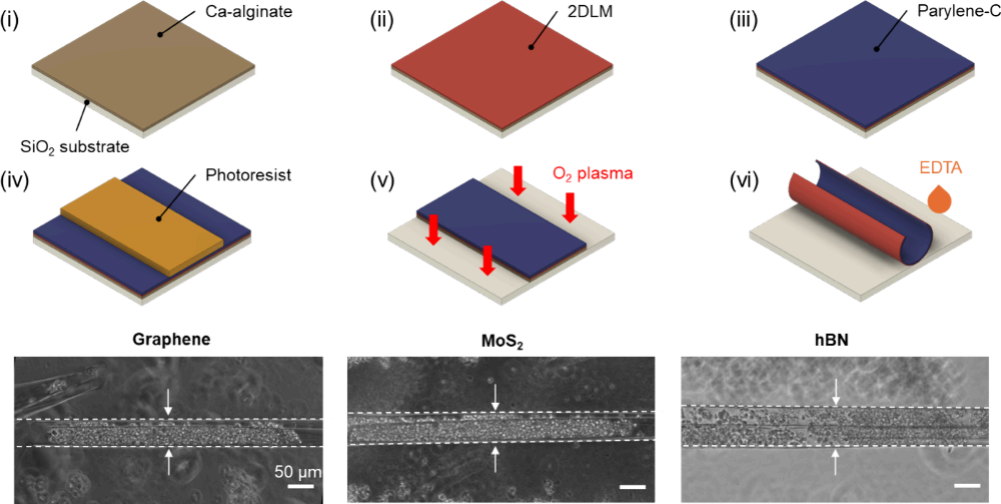

Two-dimensional layered materials (2DLMs) have received increasing attention for their potential in bioelectronics due to their favorable electrical, optical, and mechanical properties. The transformation of the planar structures of 2DLMs into complex 3D shapes is a key strategic step toward creating conformal biointerfaces with cells and applying them as scaffolds to simultaneously guide their growth to tissues and enable integrated bioelectronic monitoring. Using a strain-engineered self-foldable bilayer, we demonstrate the facile formation of predetermined 3D microstructures of 2DLMs with controllable curvatures, called microrolls. Three types of 2DLM microrolls—graphene, hexagonal boron nitride, and molybdenum disulfide—provide scaffolds to encapsulate and organize human-induced pluripotent stem cell-derived cardiomyocytes into tubular aggregates. Encapsulating cardiomyocytes in porous 2DLMs-laden microrolls allows for real-time microscopic observation and construction of precisely shaped cardiac tissues interacting with their surroundings. The ability to combine 2DLMs of diverse properties in the same structure further demonstrates the potential of this self-folding strategy for creating flexible, ultrathin bioelectronic devices that integrate seamlessly with complex biological environments, offering real-time, noninvasive monitoring of engineered tissues and organoids.

## Introduction

The development of engineered tissues and organoids represents a transformative advancement in biomedical research, offering unprecedented insights into human biology and disease.^1,2^ These are miniaturized, three-dimensional (3D) structures formed of cells mimicking the architecture and function of real organs, enabling researchers to study organ development, model diseases, and even test new therapeutics in a controlled and physiologically relevant environment.^3−5^ Unlike traditional two-dimensional (2D) cell cultures, engineered tissues and organoids provide a more accurate representation of human tissue,^6^ which is crucial for understanding complex biological processes and developing more effective treatments. To achieve this, long-term monitoring of the tissue growth, development, and response to stimuli in real-time is imperative. Traditional optical analysis methods are not sufficient for long-term monitoring of organoids and engineered tissues due to their invasiveness, risk of phototoxicity,^7^ and potential for chemical toxicity after prolonged exposure.^8^ In contrast, devices such as microelectrode arrays (MEAs) and field-effect transistor arrays (FETs) are more suitable for long-term recording, as they offer noninvasive, high-throughput, and continuous monitoring of biomolecule release,^9,10^ pH fluctuations^11,12^ and electrophysiology.^13,14^ This information is invaluable for optimizing culture conditions, assessing the impact of drug treatments, and understanding the intricacies of cellular interactions.

In recent years, two-dimensional layered materials (2DLMs) have emerged as promising candidates for next-generation MEAs and FETs due to their flexibility, high surface area-to-volume ratios, and unique electrical properties. For example, the integration of graphene into FETs has shown promising results for electrophysiology studies due to its high charge carrier mobility, chemical stability, and biocompatibility.^15,16^ Along with graphene, hexagonal boron nitride (hBN) and molybdenum disulfide (MoS_2_) are particularly important. hBN is a ∼ 5 eV wide bandgap insulator with a similar breakdown voltage to silicon dioxide, high thermal conductivity, and chemical stability, making it ideal for use as a protective layer or dielectric substrate in electronic devices.^17,18^ MoS_2_, on the other hand, is a semiconductor with a bandgap dependent on the number of layers and optoelectronic responsiveness, which makes it suitable for use in field-effect transistors and photodetectors.^19,20^ Similar to graphene, monolayer hBN and MoS_2_ exhibit >80% transparency,^21,22^ which is essential for applications that require simultaneous optical and electrical readouts. Combining them can thus lead to flexible, ultrathin integrated electronic components. However, to ensure electrical coupling with 3D-structured organoids, as well as to control the growth of engineered tissues into specific shapes, there is the need to adapt 2DLMs into 3D interfaces.^23^ Consequently, increasing efforts are turning toward exploring methods to fold or otherwise transform 2DLMs into 3D configurations, aiming to bridge the gap between advanced material properties and the intricate demands of modern biological systems. A convenient approach to realize a broad range of curvature radii and parallelization is using self-folding structures that can be transformed simultaneously into a predefined shape upon a trigger mechanism.^24^ Reported heterogeneous material substrates for folding graphene include an InGaAs/Cr bilayer,^25^ polydopamine/poly(*N*-isopropylacrylamide) bilayer,^26^ and differentially strained SU-8 layers.^27^ The latter has also been applied with MoS_2_.^28^ While 2DLMs can be passively folded on a deforming substrate, as in the examples mentioned above, they can also actively contribute to the folding mechanism. In this case, the 2DLM forms one of two differentially strained stacked layers. Such active approaches are advantageous as they only require one extra material layer in addition to the material to be folded and thus result in a simple fabrication of thin devices with small (∼100 nm) achievable curvature radii.^29^ They have been applied to graphene using SiO_2_^29^ or chemical vapor deposited polymers.^30−34^ Despite these advances, the encapsulation and organization of live cells with self-folding 2DLMs beyond graphene remains unexplored.

In this work, we extend an active approach for creating self-foldable 2DLM microscale structures, called microrolls, from graphene to MoS_2_ and hBN. We use a poly(2-chloro-*p*-xylylene) (parylene-C) interface layer as a driving force for the self-folding process, with controllable radii, and employ calcium alginate (Ca-alginate) as a sacrificial layer to facilitate the release of the structures. Unlike previous methods, which rely on aggressive chemicals,^25^ elevated temperatures,^26^ or pH changes,^29^ our approach operates at physiological conditions, making it ideal for cell encapsulation. Additionally, we employ a single polymeric interface layer to induce strain, enabling the fabrication of thin (100–200 nm) self-folding films and a simplified process. This represents an alternative to methods requiring bilayer systems or complex differential strain engineering.^25−28^ We demonstrate the applicability of our 2DLM microrolls as tissue engineering scaffolds by encapsulating and building tubular-shaped aggregates of human induced pluripotent stem cell (hiPSC)-derived cardiomyocytes (CMs). Finally, we successfully rolled and analyzed stacked layers of different 2DLMs, highlighting their potential to fabricate 3D-integrated bioelectronic devices based on 2DLMs within self-folding parylene-C structures.

## Results and Discussion

### Fabrication and Folding Mechanism of 2D Materials

**Figure 1:**
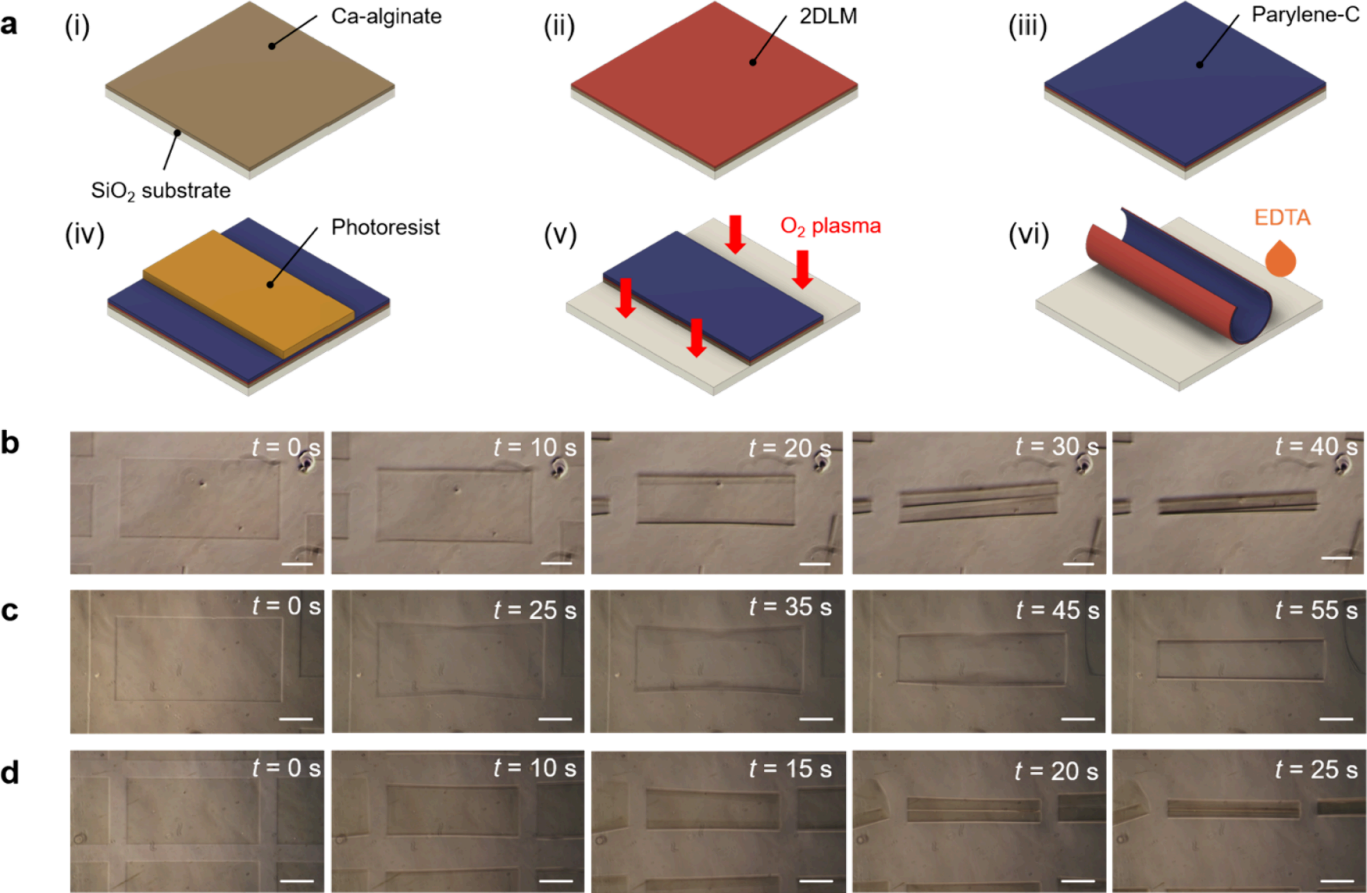
(a) Fabrication scheme of the 2DLM microrolls and time-lapse phase-contrast microscopy images of the (b) graphene, (c) hBN, and (d) MoS_2_ microrolls. Scale bars: (b) 100 and (c, d) 200 μm.

We fabricated the self-foldable 2DLM thin films following three main steps, as described in previously reported protocols (Figure 1a).^31,32,34^ First, we transferred the 2DLMs onto a SiO_2_ substrate coated with Ca-alginate via a wet transfer process. Details of the procedure can be found in the Methods section as well as in Figures S1 and S2. Next, the substrates with the 2DLMs were wholly laminated with parylene-C and micropatterned via reactive ion etching (RIE) with oxygen (O_2_) plasma through a photoresist mask. Last, we used an ethylenediaminetetraacetic acid (EDTA) solution to dissolve Ca-alginate.^35,36^ Due to the compressive strain in the parylene-C film introduced by the soft-baking of the photoresist at 90 °C, the patterned structures folded up upon dissolution of the sacrificial Ca-alginate layer. Figures 1b–d show the time-lapse images of the folding process for graphene, hBN, and MoS_2_, respectively. After adding EDTA into deionized (DI) water, the rectangular patterns started to detach and fold within a 1 min time frame (movies S1–S3). The exact duration depended on the pattern size, as the dissolution of the sacrificial Ca-alginate layer occurs gradually from the edge of the 2DLM/parylene-C bilayers.^30^ Other contributing factors to the duration were the thicknesses of the parylene-C layer and Young’s moduli of the 2DLMs, which also influenced the folding diameter of the tubes. Furthermore, the total concentration of EDTA after dilution played an important role in the dissolution rate, affecting the folding duration.^37^

**Figure 2:**
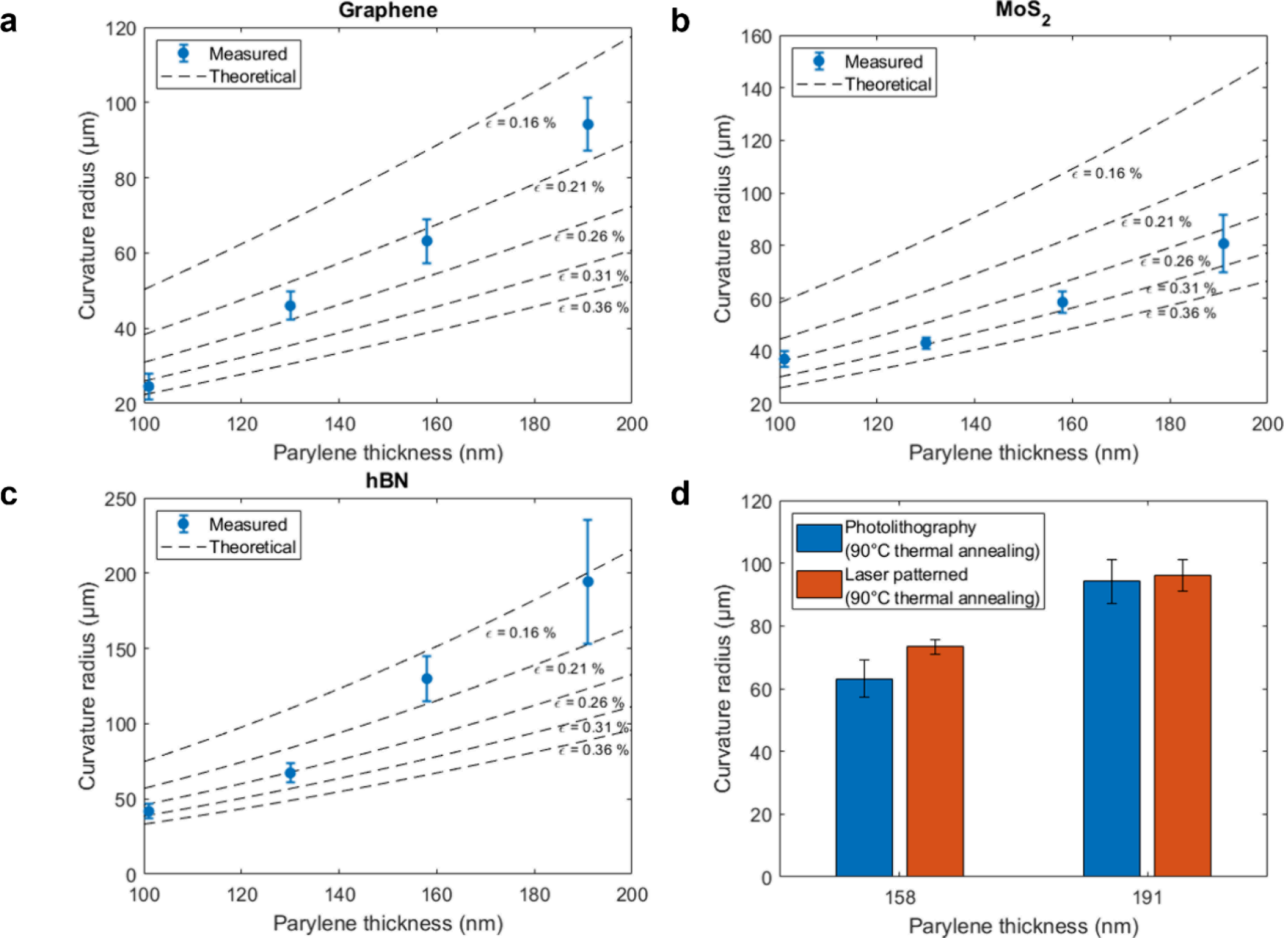
Obtained curvature radius of the (a) graphene, (b) MoS_2_, and (c) hBN microrolls dependent on the thickness of the parylene-C layer. The data points correspond to the average and standard deviation of at least 12 individual microrolls. According to (1), the theoretical predictions for a strain ratio between 0.16 and 0.36% in a 0.05% step are also plotted as a reference. (d) Comparison between the curvature radii of the photolithography patterned and the laser patterned microrolls, both thermally annealed for 2 min under 90 °C before patterning.

We investigated the final 3D architectures of the microrolls formed of each 2DLM by relating the curvature radii of the resulting tubular structures (ρ) to the parylene-C thickness (*d*_P_). We carried out quantitative predictions of ρ by applying the bimorph-beam theory,^38^ which relates ρ to *Y*, *d*, and ε as
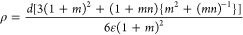
1where *d* is the total thickness of the bilayer (*d*_2D_+*d*_P_), *m* is the ratio of the bilayer thicknesses (*d*_2D_/*d*_P_), *n* is the relative elastic modulus (*Y*_2D_/*Y*_P_), and ε is the in-plane biaxial strain between the two layers. The subscript “2D” denotes the 2DLM, while “P” represents the parylene-C layer. The inserted *Y* values for graphene, MoS_2_ and hBN were 1 TPa,^39^ 270 GPa,^39^ and 220 GPa,^40^ respectively. For parylene-C, the standard value of 2.8 GPa was used. As shown in Figures 2a–c, the experimental values of ρ for the three materials largely follow the trend predicted by (1) when ε lies in the 0.16%–0.36% range. This variance in strain values compared to the ideal theoretical prediction can be attributed to the influence of the thicknesses of parylene-C in the annealing-induced strain.^41^ The estimated strains within all 2DLMs are almost in agreement with the previously reported values.^30^ Importantly, the rolled-up structures were stable, exhibiting no delamination after 4 weeks. These findings show that adhesion of 2DLMs to parylene-C is not limited to π–π stacking, as previously suggested,^30^ and indicates an important role of other surface interactions such as van der Waals forces. This mechanism of tight adhesion enhances the versatility and variety of loadable 2DLMs on the surface of parylene-C, while maintaining the ability of self-folding. Typically, the microrolls stayed rolled even after drying up (Figure S3). In some cases, however, especially when the curvature radius is large compared to the patterned structure, capillary forces between the rolls and the substrate surface may be stronger than the bending strain and lead to structure flattening.

To prove that the compressive strain in the parylene-C layer was induced during the soft bake process, we used a laser cutting machine as an alternative patterning method. In this case, the curvature radius was about 3 times larger compared to microrolls patterned with photolithography (Figure S4). The small strain observed in these samples is attributed to the intrinsic compressive strain induced in the parylene-C layer upon deposition.^42^ In contrast, when thermally annealed under the same conditions, the laser-patterned samples exhibited a curvature radius comparable to the one obtained with the original method (Figure 2d).

### Optical and Electrical Study of Rolled-Up MoS_2_

**Figure 3:**
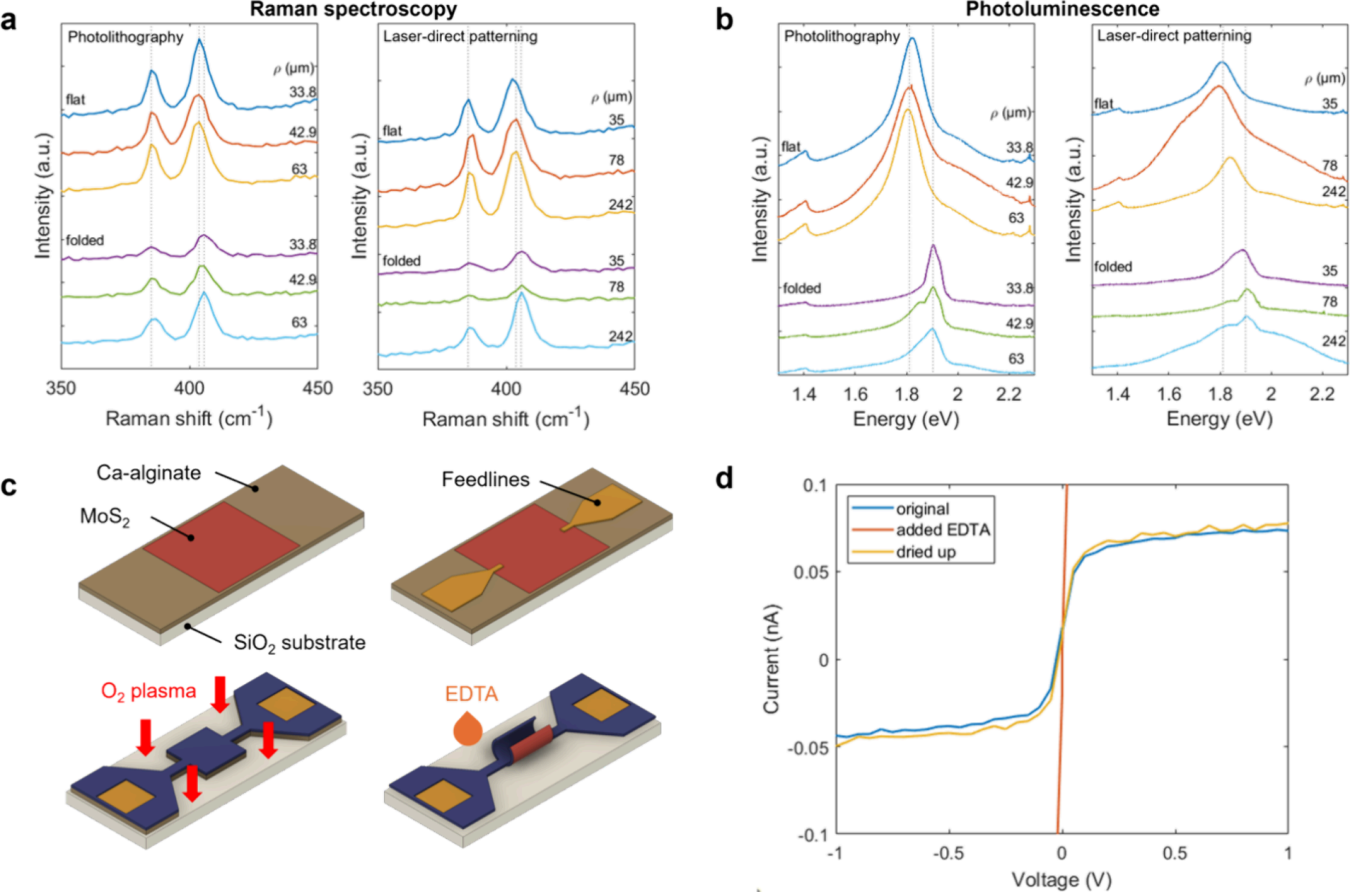
(a) Raman and (b) photoluminescence spectroscopy plots of the MoS_2_ microrolls depending on the curvature radius fabricated via photolithography (left) and laser-direct patterning (right). (c) Fabrication process of the MoS_2_ microrolls with integrated electrodes to probe the electrical properties. (d) *I*–*V* characteristics of the MoS_2_ microrolls in the original state, after adding EDTA, and after solution removal.

We used Raman spectroscopy to assess the structural changes of the 2DLMs after the self-folding process and gain insight into the strain in the 2DLM layer. An in-depth study of the graphene spectra in the flat and folded states was carried out in previous works, showing a consistent blue shift of the characteristic Raman G and 2D peaks independent of the curvature radii.^30,31^ The Raman spectra obtained from the MoS_2_ microrolls in the flat and folded state for different radii are plotted in Figure 3a. Monolayer MoS_2_ in the flat state exhibited two prominent Raman peaks: the in-plane *E’* mode at around 385 cm^–1^ and the out-of-plane *A*_1_*’* mode at around 403.8 cm^–1^. The 18–19 cm^–1^ difference between these two modes serves as a fingerprint for identifying monolayer MoS_2_.^43,44^ The *E’* mode corresponds to the in-plane vibrations of Mo and S atoms in opposite directions, while the *A*_1_*’* mode arises from the out-of-plane vibrations of only the S atoms.^45^ After the self-folding process, we observed a blue shift of 2 cm^–1^ in the *A*_1_*’* peak, while the *E’* peak remained unchanged, irrespective of the curvature radius and patterning method. While uniaxial and biaxial strains typically cause shifts in both *A*_1_*’* and *E’* peaks, the *E’* peak has been proven to be the most sensitive to both compressive and tensile biaxial strain.^46−48^ The absence of a shift in the *E’* mode is therefore associated with minimal strain. The *A*_1_*’* peak is sensitive to doping, and the observed blue shift in its position is typically associated with p-type doping of MoS_2_.^44,49,50^ Hence, the shift in the *A*_1_*’* peak can be ascribed to an effective change in the doping of the 2D membrane.

We used photoluminescence (PL) spectroscopy to study the optoelectronic properties of rolled-up MoS_2_. Figure 3b shows the PL spectrum of the MoS_2_ microrolls. We observed a shift from 1.8 to 1.9 eV compared to the flat state irrespective of the curvature radius and patterning method, indicating an increase in the optical bandgap of the material. If we attributed this shift to the compressive strain in MoS_2_ derived from the parylene-C film, the strain values would be 1 order of magnitude higher than the estimated ε values of MoS_2_ in Figure 2b (0.24%–0.3%), based on previous literature,^51−54^ contradicting the Raman measurements. One factor leading to the PL shift could be a change in the screening environment, i.e. going from a low dielectric constant environment to a very high (50 or more) dielectric constant environment. However, this effect would only contribute to a few tens of meV at most.^55^ In addition, substrates are known to induce doping on MoS_2_ due to charge traps at the sample–substrate interface;^56^ in our case, Ca-alginate most likely induces an n-doping effect. A release from the substrate would then lead to a decrease in electron concentration, bringing MoS_2_ closer to charge neutrality, where the PL emission stems primarily from neutral excitons rather than charged excitons, contributing to the observed blue shift in the PL peak.^49^ This phenomenon is consistent with the selective blue shift of the *A*_1_*’* peak and would explain the independence of the shift amount on the patterning method and curvature radius. We thus hypothesize that the scrolling of the MoS_2_-parylene-C heterostructure does not introduce any strain in the MoS_2_ monolayer and the shifts in the Raman and PL measurements are due to an interplay of dielectric constant change and a charge doping effect.

To assess the electrical properties of MoS_2_, we measured the current (*I*)-voltage (*V*) characteristics in a resistive configuration by connecting Au pads to both ends of a 300 × 800 μm^2^ MoS_2_-parylene-C bilayer film (Figure 3c). Figure 3d shows the *I*–*V* curve of MoS_2_ before and after immersion in an EDTA solution, as well as after the solution has completely dried, resulting in structure flattening due to capillary forces. The nonlinear *I*–*V* curve of MoS_2_ in the pristine state confirms the semiconductor behavior. Because of the higher resistance of MoS_2_ compared to the surrounding ionic solution, the pathway of electrons was dominantly along the EDTA solution and showed a drastic increase in conductance before reaching saturation after 5 min (Figure S5). Notably, the treatment to remove the EDTA solution and make the MoS_2_ microrolls flat again led to returning to the original *I*–*V* curve. This result verifies that the folding process as well as the immersion in ionic solution do not compromise the conductivity of MoS_2_ and are completely reversible processes.

### Tissue Engineering Scaffolds for hiPSC-CMs

**Figure 4:**
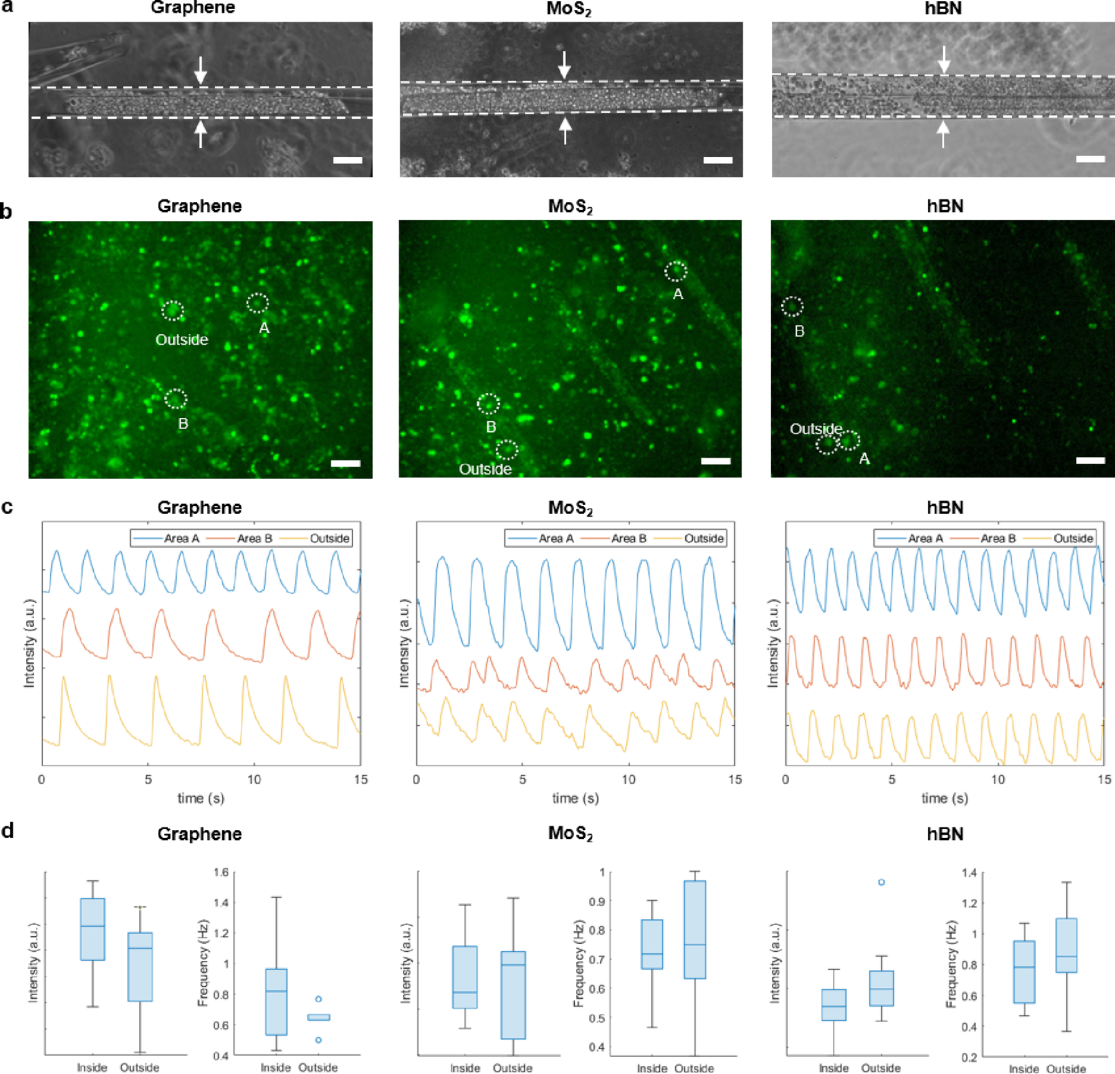
(a) Phase contrast microscopy images of the graphene, MoS_2_, and hBN microrolls encapsulating tubular-shaped aggregates of hiPSC-CMs. (b) Fluorescence images of hiPSC-CMs spheroids after Ca-imaging outside and inside the microrolls. (c) Time-dependent fluorescence intensity profiles of the spontaneous firing of the cardiomyocytes in the segments shown in (b). (d) Average intensity (left) and frequency (right) distributions of eight firing areas inside and eight firing areas outside of the graphene, MoS_2_, and hBN microrolls. In all cases, the average intensity value of a 10 × 10 pixel window ROI around the firing area was used. Scale bars: (a) 50 and (b) 100 μm.

To test the cytocompatibility of the self-folded 2DLMs, we encapsulated and investigated the physiological functions of hiPSC-CMs. We chose them for their relevance in cardiology research as well as their capability of spontaneous beating, which can be used as a biomarker for cardiotoxicity tests.^57^ We fabricated each array of graphene, MoS_2_, and hBN microrolls for this purpose. To facilitate nutrient diffusion inside the tubes, 8 × 8 μm pores were patterned every 40 μm. Because the RPMI medium of the differentiated cardiomyocytes can dissolve Ca-alginate, adding EDTA was not required for this experiment. The encapsulation of cells inside the detached microrolls was confirmed 4 h after seeding the cells on top of the structures. The cells were then stably incubated and observed for 6 days. Figure 4a depicts phase-contrast images of the hiPSC-CMs encapsulated in the graphene, MoS_2_, and hBN microrolls on day 6. It is observed that the mechanical constraints introduced by the 2DLM microrolls guided the aggregation of hiPSC-CMs into tubular structures.

We used calcium imaging to study the physiology of the hiPSC-CMs both inside and outside of the 2DLM microrolls. Figure 4b shows fluorescence images of the three materials after staining with fluorogenic calcium-sensitive dyes. In this case, we detected several small, spontaneously firing spheroids both inside and outside the microrolls, indicating the noncytotoxicity of the self-folded 2DLMs and parylene-C. The fluorescence patterns of three highlighted sample spheroids are shown in Figure 4c and movies S4–S6 for each of the three materials. A more detailed statistical analysis shows no significant difference in the average fluorescence intensity and firing rate between randomly selected spheroids inside and outside the microrolls (Figure 4d). The similar intensity inside and outside the tubes confirms the high transparency of the used materials. Barrier-free observation is a crucial advantage in functional devices as it allows for real-time optical verification of the measured signals. The comparable beating frequencies of spheroids inside and outside the tubes further imply that the cell physiology was not negatively affected by the encapsulation or by the presence of the 2DLMs. These findings demonstrate the potential of the 2DLM-laden microrolls for application as tissue engineering scaffolds while maintaining their material properties. The average peak intensities and periods for each individual firing area can be found in Figure S6.

### Multilayered 2D Materials and Insulation Quality of hBN

Finally, we tested the self-folding approach with more than one layer of 2DLM. This is important since the combination of these materials has been shown to enhance and induce new properties. For example, graphene devices supported by an hBN monolayer substrate provide enhanced mobility and carrier homogeneity, leading to a dramatic improvement in device performance.^58,59^ Furthermore, graphene–MoS_2_ bilayers were shown to generate a Schottky junction, which can be used for building self-powered photodetectors.^60^ Thanks to its insulating and passivating nature, which enhances the transport properties of graphene and MoS_2_ by protecting them from environmental contaminants.^58^ hBN can be further used as an ultrathin insulating layer between electrical components in integrated devices. We fabricate microrolls starting from heterobilayers of, top to bottom, graphene–MoS_2_, graphene–hBN, and hBN–graphene. The layer on top is the one interfacing with the parylene-C film.

**Figure 5:**
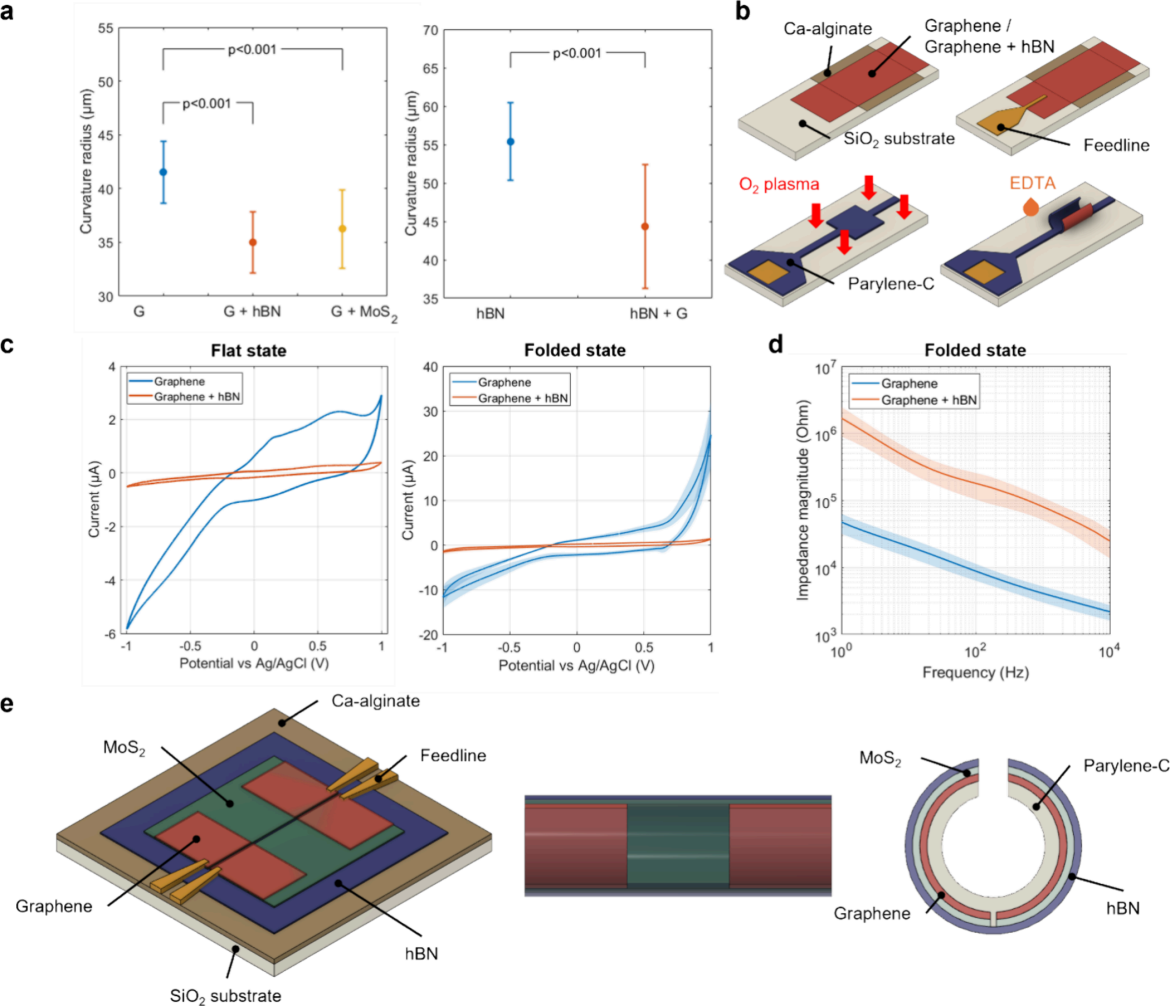
(a) Comparison between the curvature radii of microrolls with one and two layers of 2DLMs for a parylene-C thickness 115 nm. The 2DLM directly interfacing with parylene-C is graphene (left) and hBN (right). The data points correspond to the average and standard deviation of at least 12 individual microrolls. (b) Fabrication process of the graphene and graphene–hBN microrolls for electrochemical tests. (c) CV curves of graphene microrolls without and with a monolayer hBN passivation layer in the flat and folded states. For the folded state, a total of 10 channels per case were evaluated. (d) Average electrochemical impedance magnitude of 10 graphene microrolls with and without hBN passivation. (e) Future vision of a self-folded 3D FET using the parylene-based strain-building method with a graphene-MoS_2_-hBN trilayer. The Au feedlines are in contact with the graphene layer of two separated channels along the microroll.

Figure 5a compares the curvature radii of the heterobilayers. We observed that the stacking order plays an important role in the final curvature radius. This is due to the different behaviors each material has when interfaced with the parylene-C layer, as shown in Figure 2. However, a common trend in both the graphene–parylene-C and the hBN–parylene-C interfaces is that their curvature radii decrease when stacking another 2DLM layer to the bottom end. This is the result of increasing stiffness due to an increased thickness and is consistent with the theoretical model described in (1).

We assessed the insulating quality of monolayer hBN in the graphene microrolls by investigating the electrochemical properties of the graphene-electrolyte interface with and without hBN insulation. Figure 5b shows the fabrication process of the graphene and graphene-hBN microtubular electrodes. We deposited an Au feedline between the graphene and the parylene-C layer to connect the graphene from the inside. To prevent the feedline from being exposed to the electrolyte solution, we patterned the Ca-alginate layer using a parylene-C hard mask. After folding, the hBN layer remains in the outer part, thus covering the exposed graphene area from the electrolyte solution. Figure 5c depicts the cyclic voltammetry in the flat and folded states with and without hBN insulation. As expected, the maximum current decreased by more than 10 times in the graphene–hBN microrolls for both the folded and the flat state. A similar behavior can be observed in the electrochemical impedance spectrum plotted in Figure 5d, where the hBN layer increases the net impedance by more than 1 order of magnitude over the measured frequency range. The phase information on the impedance can be found in Figure S7. These findings mean that the hBN layer successfully insulates the graphene electrode. The fact that there is still a measurable current with hBN indicates that the graphene edges between the parylene-C and hBN layers are exposed or could be caused by the presence of defects in the hBN monolayer.

The multiple stacked 2DLM layers, in conjunction with their diverse electrical properties, facilitate the development of more complex, self-foldable, transparent biosensing components, such as FETs. These sensors offer simultaneous optical, electrical, and chemical monitoring of cells, significantly improving real-time, long-term tracking of engineered tissues and organoids without the drawbacks of conventional and nontransparent 2D biosensors (Figure 5e).

## Conclusions

We demonstrated controllable self-folding of monolayer MoS_2_ and hBN by simply transferring a parylene-C film on top. The lack of in-plane strain in the 2DLM layer contributed to a damage-free, stable folding of the 2DLMs. We further showed that 2DLM microrolls were capable of guiding the aggregation and maintaining the physiological functions of encapsulated hiPSC-CMs, making them suitable as tissue engineering platforms. The stable adhesion between different 2DLMs and parylene-C suggests compatibility with other 2D materials, further expanding the range of functional devices that can be built using this approach. The concept requires the use of semicrystalline polymers in which thermal annealing leads to structural changes such as increased crystallinity or stress relaxation. A CVD process for depositing the polymeric layer is also advantageous, as it maximizes interaction areas, enhancing van der Waals forces and ensuring a robust adhesion. Finally, the parylene-C-based self-folding of 2DLMs is not only limited to *in vitro* applications but could also be expanded for implants, such as self-folding bioelectronic devices for nerve cuff interfacing.

## Methods

### Chemicals

Monolayer, polycrystalline, CVD-grown graphene (100 × 100 mm^2^ square on Cu foils) and hBN (75 × 75 mm^2^ square on Cu foils), alginic acid sodium (71238, Salts from brown algae), and calcium chloride dihydrate (C8106) were purchased from Sigma-Aldrich (Merck) Germany. Atmospheric pressure chemical vapor deposited (APCVD) polycrystalline monolayer MoS_2_ sheet on SiO_2_ substrates was purchased from 2D semiconductor (CVD-MoS_2_-ML-S, Full coverage 100%, 10 × 10 mm^2^ square on SiO_2_/Si substrates). This MoS_2_ sheet possesses a hexagonal 1H phase and 1.85 eV direct bandgap. Parylene-C, positive photoresist (MICROPOSIT S1813G) and its developer (MICROPOSIT 351) were purchased from Kayaku Advanced Materials Inc. SiO_2_ substrates (thickness no. 1, 22 × 22 mm^2^, Marienfeld) were purchased from VWR Avantor. PDMS (Sylgard 184) was purchased from Dow US. Ethylenediaminetetraacetic acid (EDTA, 0.5M) was purchased from Thermo Fisher Scientific.

To obtain human induced pluripotent stem cell cardiomyocytes (hiPSC-CMs) we used hiPSC line WTC-11 (GM25256, Coriell Institute). Essential 8 Flex media for hiPSC maintenance and RPMI-1640 supplemented with B27 minus insulin for cardiac differentiation as well as Versene and Geltrex LDEV-free, hESC-qualified, reduced growth factor basement membrane matrix was purchased from Gibco, ThermoFisher Scientific. The small molecule inhibitors ROCK-inhibitor Y-27632, CHIR99021, and IWR-1-endo were bought at Stemcell Technologies. Accutase and Sodium Lactate were purchased from Sigma-Aldrich, and the tissue culture plastic was purchased from Sarstedt. The 70 μm cell strainers were bought from Corning.

### Fabrication of Self-Foldable Films with 2D Materials

Figure 1a illustrates the fabrication process of micropatterned Ca-alginate/2DLM/parylene-C trilayers. A 1 wt % sodium alginate solution was spin-coated onto 0.13–0.16 mm thick SiO_2_ wafers at a peak speed of 3000 rpm for 30 s, leading to a layer thickness of around 50 nm. The wafers were then submerged in a 100 mM calcium chloride solution to induce gelation of the Ca-alginate (i). The 2DLMs were subsequently transferred onto the Ca-alginate surface using the conventional chemical etchant-assisted wet transfer technique (ii).^61^ Specifically, the 2DLMs were first coated with a 120 nm thick layer of poly(methyl methacrylate) (PMMA), and their original CVD synthesis substrates were etched away using specific etchants. Monolayer graphene and hBN were transferred by dissolving their Cu substrates using FeCl_3_, while monolayer MoS_2_, grown on a Si/SiO_2_ substrate, was transferred by removing the SiO_2_ layer through the application of a drop of 1 M KOH solution.^62^ After the 2DLMs were transferred, the PMMA coating was eliminated by immersing the sample in acetone. Finally, the Ca-alginate/2DLMs bilayer was coated with parylene-C (iii), with a thickness ranging from 101 to 191 nm, using CVD (SCS LABCOATER PDS2010). During deposition, dichloro-di(*p*-xylylene) was vaporized at 150 °C and subsequently pyrolyzed at 690 °C to produce a chloro-*p*-xylylene monomer, which was then deposited onto the 2DLM surface to form uniform parylene-C membranes. The obtained thickness was then measured with a Bruker Dektak XT Profilometer.

We utilized photolithography or laser-based direct patterning to micropattern the trilayer consisting of Ca-alginate, parylene-C, and 2DLMs. The triple-layered film was first coated with S1813G, serving as a hard mask. The S1813G was micropatterned on the parylene-C surface through photolithography (Heidelberg Instruments μMLA), providing protection during the subsequent RIE steps (iv). The layers were then etched using reactive ion etching (RIE) with oxygen plasma (PT7170, Bio-Rad, Hercules, CA; etching gas: O_2_; etching time: 20–25 min) to produce a micropatterned film array (v). Laser patterning was conducted with a Keyence MD-U1000C Marking Laser. Finally, the arrays were released from the SiO_2_ wafer by immersing them in an aqueous EDTA solution (vi).

### Fabrication of Multichannel Devices

We fabricated the samples for *I–V* measurements (Figure 3c) on a 100 nm SiO_2_/Si substrate. After coating Ca alginate and transferring MoS_2_, we sputtered 50 nm gold feedlines using a laser patterned 75 μm thick Kapton film hard mask (CMC 70075). The feedlines connect the MoS_2_ layer from two terminals to achieve a resistive configuration. We then deposited the parylene-C layer and opened the contact pads using RIE.

The samples for electrochemical measurements (Figure 5b) were fabricated by using a laser-patterned parylene-C hard mask (5 μm thick) to spin coat the sodium alginate solution only in the areas where the microrolls are meant to fold. This way, we ensure that the feedlines will not detach and get exposed to the electrolyte solution. We then peeled off the parylene-C hard mask during the immersion in calcium chloride solution and continued with the 2D material transfer. Before the parylene-C deposition, a laser patterned 75 μm thick Kapton film hard mask (CMC 70075) was used for sputtering 50 nm gold feedlines to connect with the graphene layer from the inside. The feedlines connected with the graphene layer only from one terminal to assess the 2D material–electrolyte interface. After depositing parylene-C and patterning the photoresist mask, the contact pads were opened using RIE. Finally, a SiO_2_ ring was glued onto the sample using PDMS to protect the contact pads from the electrolyte solution. In this case, the parylene-C layer also serves as passivation for the gold feedlines. The patterned designs of the individual layers are displayed in Figure S8.

### Optical Analysis of 2DLM Microrolls

The MoS_2_-parylene-C bilayer films were analyzed using a confocal Raman microscope (Renishaw, Invia) with a 532 nm excitation wavelength and a standard grating of 1800 lines/mm. For Raman and photoluminescence analysis, the microrolls were suspended in water within glass dishes (BR455701, Sigma-Aldrich). The microrolls were observed using a 50× lens with a long working distance and a numerical aperture of 0.5. The laser power was maintained at 5 mW. Spectra ranging from 100 to 3200 cm^–1^ were recorded for Raman spectroscopy. For photoluminescence spectroscopy, the spectrum ranged from 1.3 to 2.3 eV, with each measurement accumulated twice. The measurement accuracy was confirmed against a Si reference at a wavenumber of 520 nm.

### Electrical and Electrochemical Analysis of 2DLM Microrolls

A prober station (Infineon Ag MCH) equipped with 4-probe heads (DPP 105, Cascade Microtech) and an upright microscope (Leica S6), featuring 100 μm titanium tips arranged in a straight line 1 mm apart, was used in conjunction with a digital multimeter (Keithley) to record *I* – *V* characteristics. The *I* – *V* characteristics were measured in an aqueous solution, consisting of distilled water and EDTA, at room temperature. A voltage was applied between the two terminals while the corresponding currents were recorded.

Cyclic voltammetry (CV) and electrochemical impedance spectroscopy (EIS) were employed to analyze the tubes containing graphene and graphene–hBN using a potentiostat (VSP-300, Bio-Logic Science Instruments, Seyssinet-Pariset, France). Both techniques utilized phosphate-buffered saline (Modified Dulbecco’s PBS, Sigma-Aldrich) as electrolyte. CV and EIS were conducted in a 3-electrode configuration, with an Ag/AgCl as reference and Pt wire as counter electrodes. The CV curve was obtained within a potential range of −1 to 1 V vs Ag/AgCl (3 M NaCl), with a scan rate of 100 mV s^–1^. For EIS, a sinusoidal waveform with an amplitude of 10 mV vs. the open circuit potential was used to measure the impedance of the electrodes across a frequency range of 1–10^4^ Hz.

### Cell Culture and Differentiation

hiPSCs were cultured in a humidified Thermo Heracell incubator at 37 °C and 5% CO_2_. hiPSCs were maintained in Essential 8 (E8) Flex media until they reached a confluency of 75%–85%. Cells were rinsed with PBS once and then incubated with 1 mL Versene for 4 min at 37 °C. Versene was aspirated, and 1 mL E8 flex media with 5 μM Rock inhibitor Y-27632 was used to carefully detach the cells by pipetting. Cells were resuspended with a splitting ratio of 1:10 in E8 Flex media with 5 μM Y-27632 and seeded on prepared Geltrex-coated plates. Tissue culture plates were coated according to the manufacturer’s protocol. The media was replaced the following day to remove Y-27632 and then every 48 h until the confluency for passaging was reached.

To obtain hiPSC-CMs, we followed the GSK3 inhibitor and Wnt inhibitor (GiWi) differentiation protocol.^63^ hiPSCs were passaged as described above and were seeded into 12 well plates for cardiac differentiation at a cell density of 100.000 cells/well. The rock-inhibitor was removed the following day. When cells reached a confluency of 50–60% (Day 0), cardiac differentiation was induced by adding 6 μM CHIR99021 in RPMI/B27 minus insulin for 48 h. After 48 h, the media was replaced by RPMI/B27 minus insulin, and on the next day, 5 μM IWR-1-endo in RPMI/B27 minus insulin was added for 48 h. On day 5 of the protocol, IWR-1-endo was removed and the media was replaced with RPMI/B27 minus insulin. RPMI/B27 minus insulin media was replaced every 48 h until day 9, when a lactate-based purification was applied to select cardiomyocytes. We added 0.4 mM lactate to the culture media for 48 h and, on day 11, replaced the media with glucose-free RPMI-1640 with 0.4 mM lactate for 48 h. Afterward, cells were cultured in RPMI/B27 minus insulin for another 48 h. For seeding the hiPSC-CM on the self-foldable thin films, hiPSC-CMs were rinsed once with PBS and then incubated with 1 mL Accutase for 30 min. Accutase was neutralized with 1 mL RPMI/B27 minus insulin with 5 μM Y-27632. Cells were pooled in a 50 mL conical tube and then passed through a 70 μm cell strainer to receive single cells. Cells were centrifuged at 300 g for 5 min and then resuspended in RPMI/B27 minus insulin with 5 μM Y-27632 at the desired cell density. The rock inhibitor was removed the next day by replacing the media with RPMI/B27 minus insulin. Media changes were performed every 48 h.

To provide evidence of the efficiency of the differentiation protocol, we characterized the population of cardiomyocytes using immunocytochemistry and flow cytometry. The results are summarized in Figure S9. The beating of the differentiated cardiomyocytes is observable in movie S7.

### Calcium Imaging

Live hiPSC-CMs were stained with Cal-520 AM (AAT Bioquest) to monitor changes in calcium ion flux and the associated signal propagation through the cells. The calcium indicator has excitation and emission wavelengths of 492 and 515 nm, respectively. The dye’s stock solution was prepared according to the manufacturer’s instructions. Specifically, 3 μL of 2 mM Cal-520 AM aliquots were dissolved in dimethyl sulfoxide (DMSO, Sigma-Aldrich) and stored at −20 °C. From this stock, a 1 mL working solution of 6 μM Cal-520 was prepared in the cell medium with 0.04% Pluronic F-127 (Sigma-Aldrich). Pluronic F-127, a nonionic surfactant, aids in solubilizing the hydrophobic AM ester of the fluorescent dyes like calcium, enhancing their uptake by the cells. The existing cell medium was discarded and replaced with the working solution, followed by incubation at 37 °C for 3 h. Finally, the working solution was replaced with a prewarmed supplemented medium prior to imaging. The calcium signals were recorded using a fluorescence microscope (BZ-X810, Keyence) with stage-top incubator (Tokai Hit). For analyzing the fluorescence signals, the average intensity of a 10 × 10 pixel window around the firing areas was evaluated over time. The total intensity of a firing area corresponds to the average of the firing signal over time and the firing frequency to the dominant frequency component obtained using the Fast Fourier Transform.

## Supporting Information Available

(Figure S1) Chemical etchant assisted wet transfer process of graphene and hBN using PMMA as a protective layer; (Figure S2) chemical etchant assisted wet transfer process of MoS_2_ using PMMA as a protective layer; (Figure S3) MoS_2_ microrolls after completely drying up inside DI water and EDTA solution; (Figure S4) comparison between the curvature radii of the photolithography patterned and the laser patterned microrolls without thermal annealing; (Figure S5) (a) *I*–*V* characteristics of the MoS_2_ microrolls in the original state, immediately after adding EDTA and after 5 min of immersion in EDTA solution, (b) zoomed-in version of the *I*–*V* characteristics in (a); (Figure S6) average peak intensity and period of each individual firing area over a 1 min time frame; (Figure S7) electrochemical impedance spectroscopy (EIS) of graphene microrolls with and without hBN passivation (*n* = 10) including the phase information; (Figure S8) fabrication steps of multichannel devices for electrochemical characterization; (Figure S9) characterization of cardiac cell differentiation (PDF)
(Movie S1) Self-folding process of hBN microrolls upon EDTA addition (MP4)
(Movie S2) Self-folding process of MoS_2_ microrolls upon EDTA addition (MP4)
(Movie S3) Comparison between the parylene-C patterns with (left) and without MoS_2_ layer (right) upon EDTA addition (MP4)
(Movie S4) Calcium imaging recording example of the graphene microrolls with hiPSC-CMs (MP4)
(Movie S5) Calcium imaging recording example of the hBN microrolls with hiPSC-CMs (MP4)
(Movie S6) Calcium imaging recording example of the MoS_2_ microrolls with hiPSC-CMs (MP4)
(Movie S7) Differentiated hiPSC-CMs on Day 14 of the cardiac differentiation protocol in a 12-well plate (MP4)
