# Supplementary material for: Self-Foldable Three-Dimensional Biointerfaces by Strain Engineering of Two-Dimensional Layered Materials on Polymers
Source: ACS Appl Mater Interfaces. 2025 Jan 29;17(7):10305–15. doi: 10.1021/acsami.4c17342 (PMC11843539; doi:10.1021/acsami.4c17342)
Supplement: Supplementary file 1 — am4c17342_si_001.pdf [file am4c17342_si_001.pdf]

SUPPORTING INFORMATION

Self-foldable 3D biointerfaces by strain engineering of 2D layered materials on polymers

*Alonso Ingar Romero,[1,2,] Teodora Raicevic,[1] George Al Boustani,[1,2] Mrinalini Gupta,[1] Ann-Caroline Heiler,[3] Lukas Bichlmaier,[2,3] Matteo Barbone,[1] Markus Becherer,[1] Daisuke Kiriya,[4] Shigeyoshi Inoue,[2,3] Joe Alexander,[2] Kai Müller,[1] Andreas R. Bausch,[3] Bernhard Wolfrum,[1,2] Tetsuhiko F. Teshima[*1,2,5]*

AUTHOR AFFILIATIONS

[1] School of Computation, Information and Technology, Technische Universität München, 85748 Garching, Germany

[2] Medical & Health Informatics Laboratories, NTT Research Incorporated, Sunnyvale, CA, 94085 USA

[3] School of Natural Sciences, Technische Universität München, 85748 Garching, Germany

[4] Graduate School of Arts and Sciences, The University of Tokyo, Tokyo, 153-8902, Japan

[5] Faculty of Science and Technology, Keio University, Yokohama, Kanagawa, 223–8522 Japan

* Corresponding authors: tetsuhiko.teshima@ntt-research.com

Transfer process of 2DLMs

As both graphene and hBN are grown on copper foil using CVD, we used the same transferring process as illustrated in Figure S1. First, thermal release tape is applied to the underside of the copper, and a 120 nm layer of PMMA solution is spin-coated onto the 2D material at a maximum speed of 3000 rpm for 30 s. The tape prevents wrinkles that could form due to the vacuum during spin-coating, while the PMMA serves as protection during the copper etching process. After coating, the sample is heated at 110˚C for 3 min to remove the solvent (anisole) and detach the tape. Finally, the copper with the 2D material and PMMA is immersed in a 45% $FeCl_3$ solution for etching.

MoS_2, also grown via CVD, is deposited on a Si/SiO_2 substrate, so we used a different process shown in Figure S2. Following PMMA spin-coating at 3000 rpm for 30 s, the sample is cut into four pieces. Droplets of 1 mM KOH are then applied to the edges to dissolve the SiO_2 layer. Once the MoS_2 detaches, DI water is added to lift the material to the surface and dilute the KOH.

After detaching the 2DLM from its growth substrate, it is washed twice with DI water and transferred onto a Ca-Alginate-coated substrate. The sample is dried overnight and then immersed in acetone for 24 hours to remove the PMMA.

Immunocytochemistry

WTC-11 iPSCs and iPSCs-CM, which express mEGFP-ACTN2 (AICS-0075-085, Allen Cell Collection), were fixed using 4% paraformaldehyde (Sigma-Aldrich) in PBS for 15 min at room temperature. Following three cycles of rinsing with PBS, the culture was treated with a solution of 0.1 wt% TritonX 100 (Sigma-Aldrich) in PBS for 10 min at room temperature to enhance the permeability of the cell membrane. The cells were blocked with 5 wt% BSA in PBs for 2 h at room temperature. The sample was then rinsed with PBS and an Oct4 antibody (BD Bioscience, Catalog no. 611202, 1:400 dilution) was added as the primary antibody. The primary antibody was incubated at 4°C overnight. The sample was then rinsed three times with PBS. Thereafter, the Alexa Fluor™ 555 donkey anti-mouse secondary antibody (Invitrogen, Catalog no. A-31570, dilution 1:1000) was added to the sample for 2 h at room temperature. The sample was rinsed three times with PBS. To visualize nuclei, a solution of 4 μg ml^{-1} DAPI (Sigma-Aldrich) was added to the culture. After a final three cycles of rinsing with PBS the sample was imaged using MICA confocal microscope (Leica) with a 20x/0.75 CS2 DRY objective. The results on Day 14 of cardiac differentiation are illustrated in Figure S9a.

Flowcytometric analysis

The efficiency of the cardiac differentiation protocol was quantified by differentiating WTC-11 iPSCs mEGFP-ACTN2 (AICS-0075-085, Allen Cell Collection) into cardiomyocytes, as described in the Methods section. On Day 14 of the cardiac differentiation protocol, cells were detached by incubating with 1 mL Accutase per well for 30 min at 37°C. The cells were centrifuged at 300 g for 5 min, after which they were resuspended in PBS with 1 % FBS and 0.2 mM EDTA

($1x10^6$ cells mL^{-1}). To ensure a single cell suspension, the samples were filtered through a 35 μm cell strainer into a 5 mL tube. Cell viability was determined with SytoxRed Dead Cell stain (Invitrogen). An isotype control was prepared as described above. The samples were analyzed using BD Bioscience FACSAria Fusion. In order to set gates, non-fluorescent controls and single-stained samples were used. Frontward and Sideward scatter was used to exclude cell debris and doublets. Data were acquired using FACS Diva™ software. Data were further analyzed using FlowJo V10. Figure S9b shows the results of the flow cytometry analysis of alpha-actinin expression on hiPSC-CM on Day 14 of cardiac differentiation and isotype control.

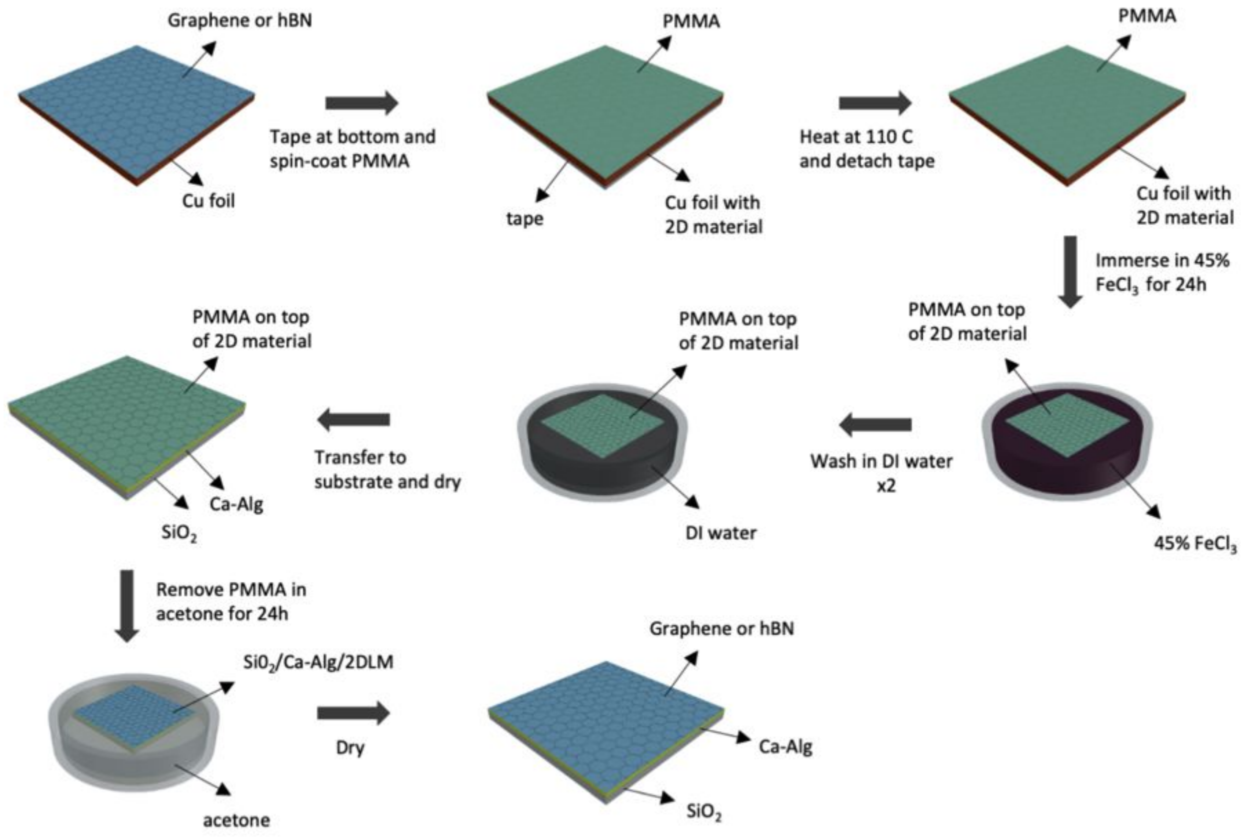

Figure S1. Chemical etchant assisted wet transfer process of graphene and hBN using PMMA as a protective layer.

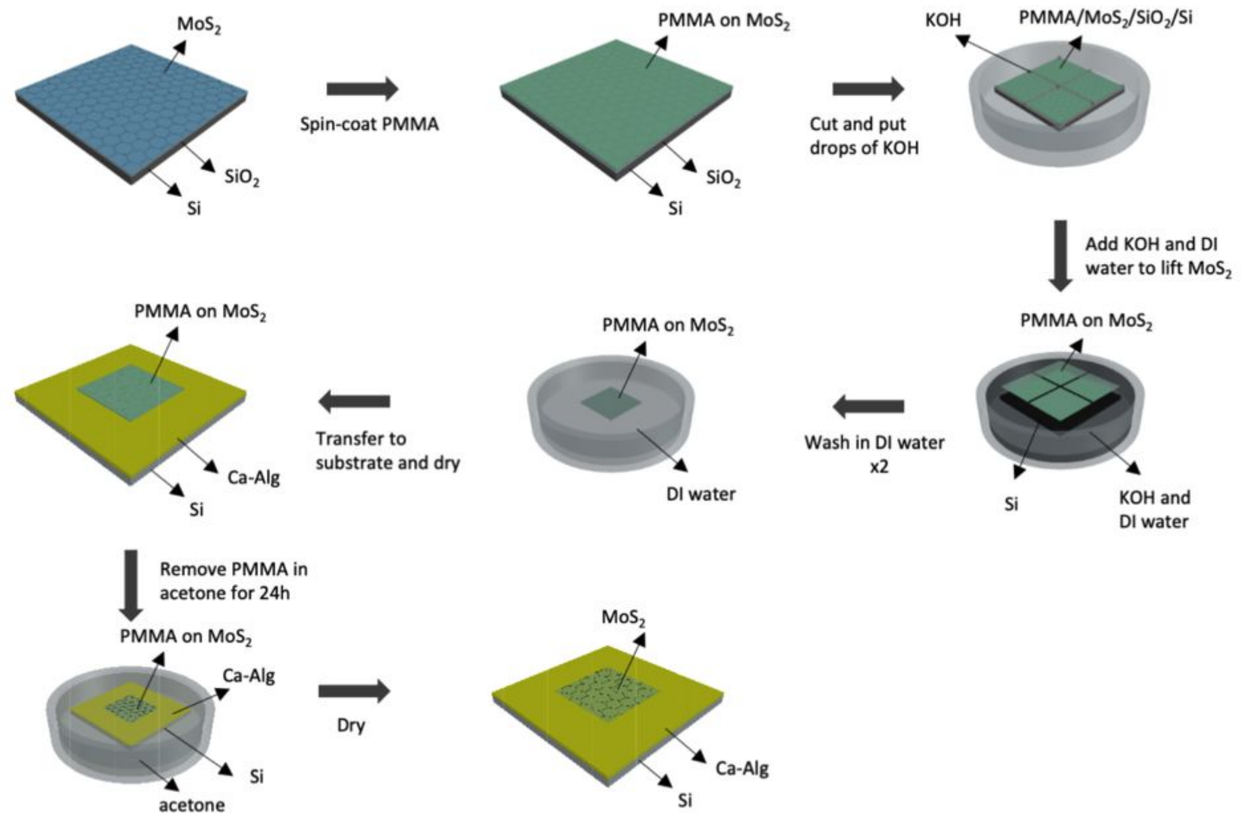

Figure S2. Chemical etchant assisted wet transfer process of MoS_2 using PMMA as a protective layer.

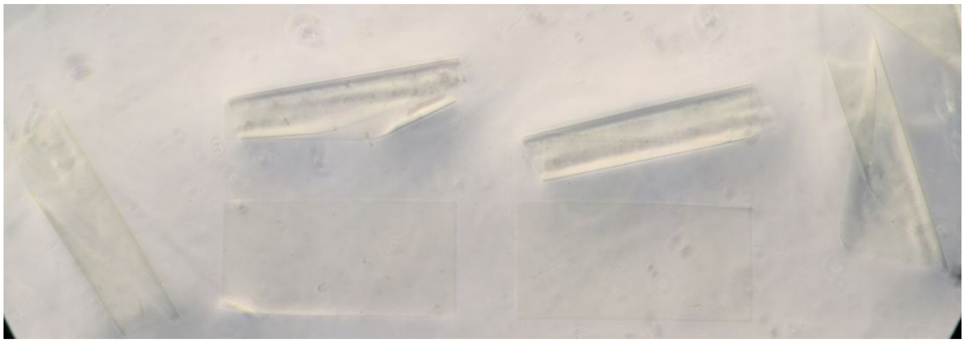

Figure S3. MoS_2 micro-rolls after completely drying up inside DI water and EDTA solution.

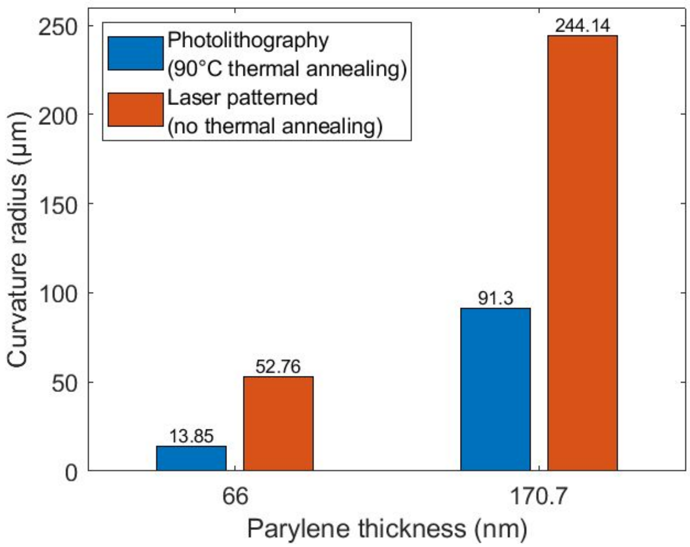

Figure S4. Comparison between the curvature radii of the photolithography patterned and the laser patterned microrolls without thermal annealing.

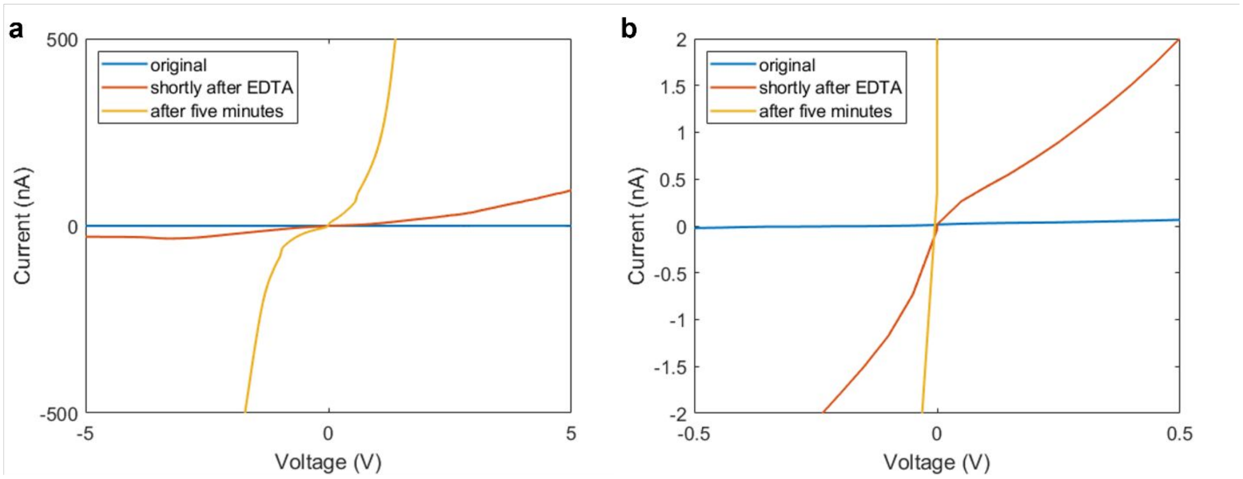

Figure S5. (a) *I*– *V* characteristics of the MoS_2 microrolls in the original state, immediately after adding EDTA and after five minutes of immersion in EDTA solution. (b) Zoomed-in version of the *I*– *V* characteristics in (a).

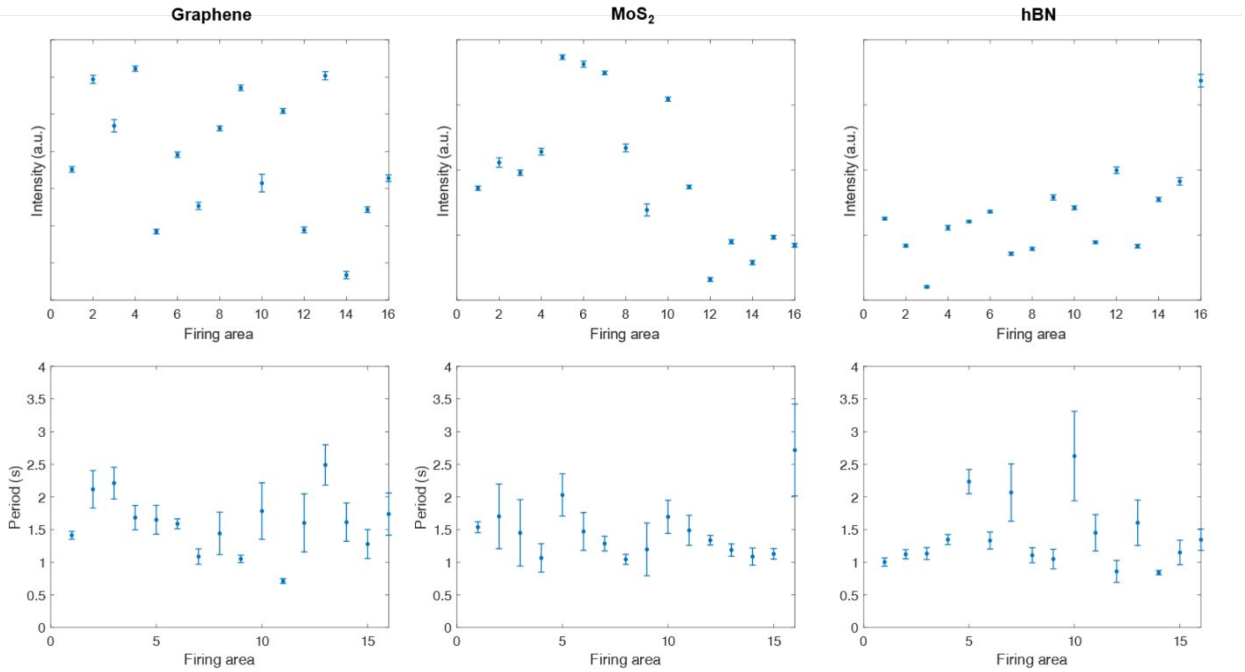

Figure S6. Average peak intensity and period of each individual firing area over a one-minute time frame. The error bars represent the corresponding standard deviations.

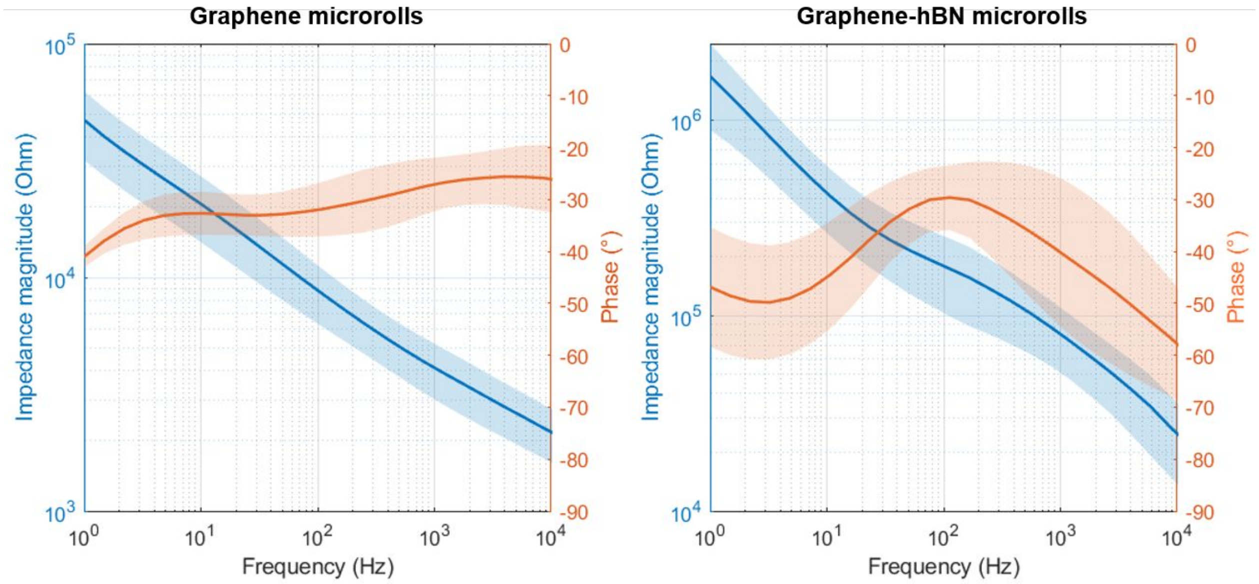

Figure S7. Electrochemical impedance spectroscopy (EIS) of graphene microrolls with and without hBN passivation (n=10) including the phase information.

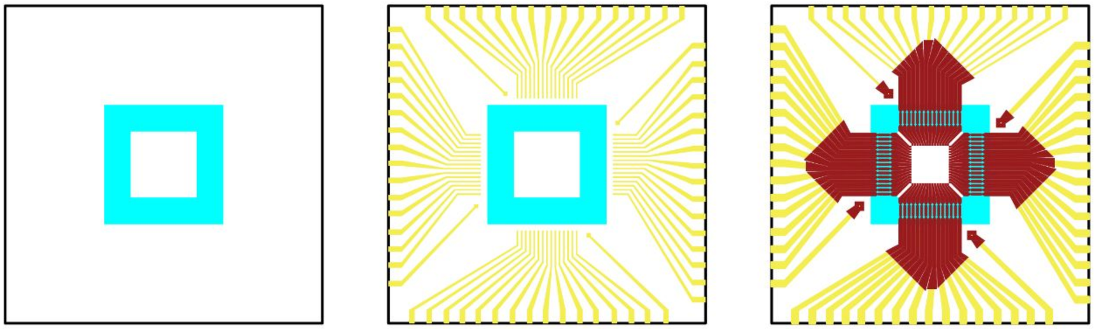

Figure S8. Fabrication steps of multichannel devices for electrochemical characterization. The three displayed layers correspond to Ca alginate (cyan), Au feedlines (yellow) and 2DLM-polymer bilayer (dark red). The sacrificial layer was patterned so that the feedlines do not get exposed to the electrolyte solution.

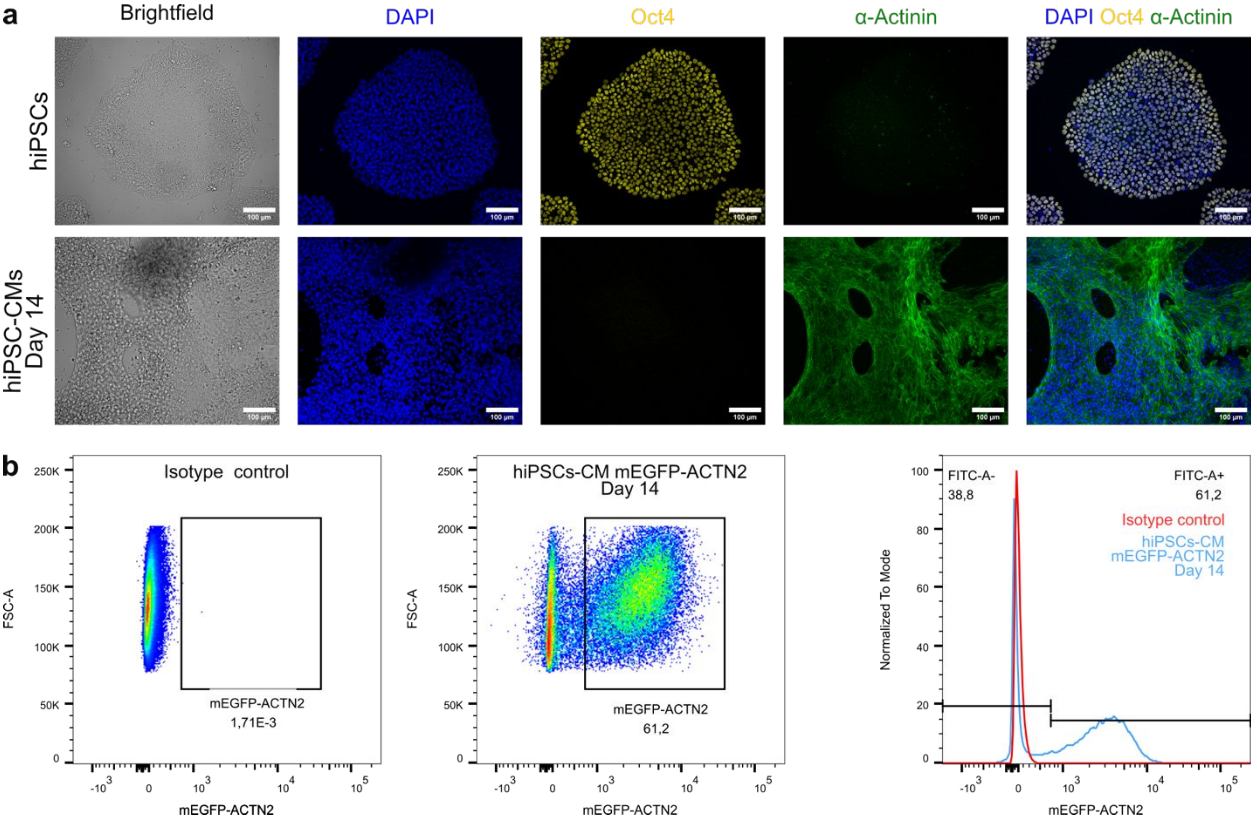

Figure S9. Characterization of cardiac cell differentiation. (a) Immunofluorescent staining for Oct4 and expression of EGFP-labelled alpha-actinin on undifferentiated iPSCs and hiPSC-derived cardiomyocytes on Day 14 of cardiac differentiation. (b) Quantitative flow cytometry analysis of alpha-actinin expression on hiPSC-CM on Day 14 of cardiac differentiation and isotype control. The blue histogram represents ACTN2-EGFP expression, and the red histogram shows the isotype control. Flow cytometric analysis confirms 61.2% viable cardiomyocytes in cell population.

ATTACHED CONTENT

movie S1. Self-folding process of hBN microrolls upon EDTA addition. (mp4)

movie S2. Self-folding process of MoS_2 microrolls upon EDTA addition. (mp4)

movie S3. Comparison between the parylene-C patterns with (left) and without MoS_2 layer (right) upon EDTA addition. (mp4)

movie S4. Calcium imaging recording example of the graphene microrolls with hiPSC-CMs. (mp4)

movie S5. Calcium imaging recording example of the hBN microrolls with hiPSC-CMs. (mp4)

movie S6. Calcium imaging recording example of the MoS_2 microrolls with hiPSC-CMs. (mp4)

movie S7. Differentiated hiPSC-CMs on Day 14 of the cardiac differentiation protocol in a 12-well plate. (mp4)
